# BCMA (TNFRSF17) Induces APRIL and BAFF Mediated Breast Cancer Cell Stemness

**DOI:** 10.3389/fonc.2018.00301

**Published:** 2018-08-07

**Authors:** Vasiliki Pelekanou, George Notas, Paraskevi Athanasouli, Konstantinos Alexakis, Fotini Kiagiadaki, Nikolaos Peroulis, Konstantina Kalyvianaki, Errika Kampouri, Hara Polioudaki, Panayiotis Theodoropoulos, Andreas Tsapis, Elias Castanas, Marilena Kampa

**Affiliations:** ^1^Laboratory of Experimental Endocrinology, School of Medicine, University of Crete, Heraklion, Greece; ^2^Department of Biochemistry, School of Medicine, University of Crete, Heraklion, Greece

**Keywords:** APRIL (TNFSF13), BAFF (TNFSF13B), BCMA (TNFRSF17), pluripotency, cancer stem cells, therapy resistance, aromatase inhibitors, androgen

## Abstract

Recent advances in cancer immunology revealed immune-related properties of cancer cells as novel promising therapeutic targets. The two TNF superfamily members, APRIL (TNFSF13), and BAFF (TNFSF13B), which are type II membrane proteins, released in active forms by proteolytic cleavage and are primarily involved in B-lymphocyte maturation, have also been associated with tumor growth and aggressiveness in several solid tumors, including breast cancer. In the present work we studied the effect of APRIL and BAFF on epithelial to mesenchymal transition, migration, and stemness of breast cancer cells. Our findings show that both molecules increase epithelial to mesenchymal transition and migratory capacity of breast cancer cells, as well as cancer stem cell numbers, by increasing the expression of pluripotency genes such as ALDH1A1, KLF4, and NANOG. These effects are mediated by their common receptor BCMA (TNFRSF17) and the JNK signaling pathway. Interestingly, transcriptional data analysis from breast cancer cells and patients revealed that androgens can increase APRIL transcription and subsequently, in an autocrine/paracrine manner, enhance its pluripotency effect. In conclusion, our data suggest a possible role of APRIL and BAFF in breast cancer disease progression and provide evidence for a new possible mechanism of therapy resistance, that could be particularly relevant in aromatase inhibitors-treated patients, were local androgen is increased.

## Introduction

Breast cancer 5-year survival has significantly increased, due to early detection and the introduction of therapies, such as specific estrogen receptor modulators, aromatase inhibitors, specific chemotherapeutic agents and more comprehensive recent precision medicine strategies. However, long (10-years) survival decreases dramatically to 78% (http://www.cancerresearchuk.org/health-professional/cancer-statistics/statistics-by-cancer-type/breast-cancer/survival#heading-Zero), suggesting that, in spite of our progress in breast cancer cell biology, other biological elements and mechanisms are involved, which remain to be uncovered or understood.

In the normal mammary gland [see (1), for a recent review] and in various mammary pathologies (2), adult stem cells have been identified to participate in its development and function. On the other hand, it is widely accepted that cancer stem cells are key players in tumor development, progression, and metastasis, as well as in tumor resistance to therapy. This cell population displays potential to proliferate infinitely, to initiate and/or reform a tumor and to induce an enhanced resistance to therapy (3). Indeed, breast cancer stem cells interact in their niche with surrounding stromal and epithelial cells, through specific molecular crosstalk pathways (4, 5). Additionally, cancer-immune cell interactions (such as PD-L1 and PD1 ligation on tumor and T cells respectively) also occur, mostly orchestrated by the tumor microenvironment (6, 7).

Until now, all established immune-related therapies target immune cells (resident or infiltrating the tumor stroma) (8), leading to an immune checkpoint blockade (9), while the notion of immune-related properties of the cancer cell *per se* and its regulation as a possible therapeutic target is less well-defined (10, 11). Several immune-related molecules are involved in immune interactions and being targeted in tumor immunotherapy approaches. Among them, TNF superfamily members (including TNF, FAS, and TRAIL and their receptors) (8), have been actively investigated and targeted in a number of malignancies. However, the TNF superfamily includes 19 different ligands and 29 receptors, which control cell survival and differentiation and plays an important role in the growth, organization, and homeostasis of different tissues, by modulating major signaling pathways (12).

Our group has focused on two members of this superfamily, whose role in cancer is less well-defined, namely APRIL (A PRoliferation Inducing Ligand, TNFSF13) and BAFF (B-cell Activating Factor of the TNF Family, also known as B Lymphocyte Stimulator (BLyS), TNFSF13B). These two ligands, act via two common receptors, B-Cell Maturation Antigen (BCMA, TNFRSF17), and Transmembrane Activator, and CAML Interactor (TACI, TNFRSF13B), while additionally, BAFF-Receptor (BAFF-R, TNFRSF13C) is a specific receptor for BAFF. They had initially been reported to exert a pivotal role in lymphocyte maturation; however, they have been also identified as significant players in several other conditions, including neoplasia (13). BAFF and APRIL have been detected in different solid tumors (14). They can activate kinase signaling pathways, such as p38, JNK or NFκB and to induce, in the majority of cases, cell survival and growth. Previously, we have shown BAFF and/or APRIL presence in many normal tissues and solid tumors, including breast cancer (15–17). BAFF is constantly expressed in tumors, while APRIL is related to breast cancer tumor grade (15). Recently, higher APRIL expression was shown in human triple negative carcinomas and APRIL was reported to induce cell proliferation both *in vitro* and *in vivo*, suggesting an association of APRIL signaling pathways with tumor aggressiveness (18). In the present work, we investigated the possible role of APRIL and BAFF in inducing breast cancer stemness, and the possible relationship of APRIL and BAFF with sex steroids, important players in breast cancer evolution and therapy. Our findings revealed that androgens, acting in an extranuclear manner, enhance APRIL transcription and subsequently its pluripotency-initiating effect.

## Material and methods

### Cell cultures and chemicals

The T47D (a luminal A type cell line: ERα+, PR+, and HER2-) and MDA-MB-231 (basal type cell line: triple negative) breast cancer cell lines were purchased from DSMZ (Braunschweig, Germany), were tested as mycoplasma free and were cultured in RPMI supplemented with 10% fetal bovine serum (FBS), at 37°C, 5% CO_2_. All media were purchased from Invitrogen (Carlsbad, USA) and all chemicals from Sigma (St. Louis, MO) unless otherwise stated.

### RT-PCR

RT-PCR was performed as described previously (19). Total RNA was isolated with the Nucleospin RNA II isolation kit (Macherey-Nagel EURL, Fr). The absence of DNA was verified by PCR for G3PDH. One μg of RNA was subjected to ABI High Capacity cDNA reverse transcription kit (Applied Biosystems, Foster City, CA). Positive controls were run in parallel with samples included in the study. We used adipose-derived mesenchymal stem cells as positive controls of BAFF and APRIL (16), isolated human lymphocytes as positive controls for BAFFR and TACI, and HepG2 cells for BCMA (19). We have used the following primers: **BAFF** forward: 5′-TTC TAG GGC ACT TCC CCT TT-3′, reverse: 5′-CTC AAG ACT GCT TGC AAC TGA-3′; **APRIL** forward: 5′-TCT CCT TTT CCG GGA TCT CT-3′, reverse: 5′-CCA GAA TGG GGA AGG GTA TC-3′; **BAFFR** forward: 5′-AGG ACG CCC CAG AGC C-3′, reverse: 5′-AGT GTC TGT GCT TCT GCA GG-3′; **TACI** forward: 5′-AGT GAA CCT TCC ACC AGA GC-3′, reverse: 5′-CTC TTC TTG AGG AAG CAG GC-3′; **BCMA** forward: 5′-GTC AGC GTT ATT GTA ATG CAA GTG T-3′, reverse: 5′-TCT TTT CCA GGT CAA TGT TAG CC-3′; **18S** RNA forward: 5′-ATG GTC AAC CCC ACC GTG T-3′, reverse: 5′-TTC TGC TGT CTT TGG AAC TTT GTC-3′. All primers were selected from qPrimer Depot, recently replaced by PrimerBank (https://pga.mgh.harvard.edu/primerbank/) and synthesized by VBC Biotech (Vienna, Austria).

### Quantitative real time PCR

Total RNA was isolated with the Nucleospin RNA II isolation kit (Macherey-Nagel EURL, Fr). The absence of DNA was verified by PCR for G3PDH. One μg of RNA was subjected to ABI High Capacity cDNA reverse transcription kit (Applied Biosystems, Foster City, CA). Real-time PCR with SYBR Green was performed with DyNAmo SYBR Green qPCR Kit (Finnzymes, Oy, Finland), using the StepOnePlus™ System (Applied Biosystems), at 95°C for 3 min followed by 40 cycles of 95°C for 15 s, 60°C for 30 s, 72°C for 60 s. PCR reactions were performed using the following primer pairs (synthesized by Eurofins Genomics): **c-MYC**: forward CAC CGA GTC GTA GTC GAG GT and reverse TTT CGG GTA GTG GAA AAC CA **SOX-2**: forward AGG AGC CTT CCT TTT CCA GA and reverse CGA CGA GAG CTC CTA CCA AC, **ALDH1A1**: forward TCC TCC TCA GTT GCA GGA TT and reverse GCA CGC CAG ACT TAC CTG TC, **KLF4**: forward GTC AGT TCA TCT GAG CGG G and reverse AGA GTT CCC ATC TCA AGG CA. The number of cycles used was optimized to fall within the linear range of PCR amplification. Changes were normalized according to cyclophilin A expression (forward: ATG GTC AAC CCC ACC GTG T and reverse: TTC TGC TGT CTT TGG AAC TTT GTC).

### Human transcriptome data analysis

Data from GSE18146 (testosterone and testosterone-BSA treated T47D and MDA cells) and GSE32666 and GSE32668 (estradiol and Estradiol-BSA treated T47D and MDA cells respectively) were downloaded from the GEO web resource as normalized series matrix files and the corresponding values of APRIL (TNFSF13) and BAFF (TNFSF13B) were extracted. The conditions and treatments are detailed in references (20, 21).

Normalized data matrix of 56 breast cancer cell line microarray analysis (22) was downloaded from the Array Express server (https://www.ebi.ac.uk/arrayexpress, accession number E-TABM-157) and corresponding co-expression values were analyzed with Origin 8 (OriginLab co, Northampton, MA).

Analysis of co-expression of APRIL (TNFSF13), BAFF (TNFSF13B), BCMA (TNFRSF17), androgen receptor (AR) and pluripotency genes ALDH1A1, SOX2, and KLF4 in the 2509 samples of the MetaBRIC study (23, 24) through the web resource cBioPortal for Cancer Discovery (25–27). From the same environment we downloaded data of APRIL expression in different molecular (PAM50+claudin and 3-gene) and ER-IHC classification, which were further analyzed for statistical significance with one-way ANOVA and *post-hoc* group comparisons (Turkey correction) in Graph Pad Prism V6 for Windows.

Finally, from the GEO archive (https://www.ncbi.nlm.nih.gov/gds/), GDS3116 study (28, 29) was identified with paired transcriptome data of 53 letrozole (an aromatase inhibitor, AI) treated patients, prior and 14 days following AI therapy together with their response to treatment.TNFSF13 (APRIL) and TNFSF13B (BAFF) data, together with the corresponding clinical information were downloaded through the NCBI-GEO online analysis tool, and analyzed by Graph-Pad Prism V 6. Response was estimated by changes in estrogen responsive genes at 14 days and confirmed by ultrasound-detected changes in tumor volume (>50% after 3 months of treatment). 37/53 (69.8%) patients responded to the therapy, while 16/53 (30.1%) were non-responders. This cohort was further analyzed for transcription factor changes with the Web resource ISMARA (30), which predicts transcription factor modifications through gene transcript changes.

### Detection of cancer stem cells

#### Autofluorescence based detection

Cells, after treatment with APRIL or BAFF (100 ng/ml) for 4 days, were detached by trypsin-EDTA from the culture plate and centrifuged (800 g 10 min). The pellet was re-suspended in PBS+2% FBS, at a concentration of 1 × 10^6^ cells/ml. They were analyzed by flow cytometry (Attune® Acoustic Focusing Cytometer, Applied Biosystems) at a cell population of at least 20,000 at 488 (580/30)/488(530/40 BL2-A/BL1-A) Dot Blot Diagram, according to Miranda-Lorenzo and colleagues, that cancer stem cells exhibit a higher level of autofluorescence (31).

#### Aldehyde dehydrogenase activity-based detection

Stem cells, that have the characteristic to express high levels of the enzyme aldehyde dehydrogenase (ALDH) were detected by the use of ALDEFLUOR™ kit (Stem Cell Technologies Inc., Vancouver, Canada). According to the manufacturer's instructions, a cell suspension of 1 × 10^6^ cells/ml assay buffer, untreated or after treatment with APRIL or BAFF (100 ng/ml) for 4 days, was incubated (45 min, at 37°C) with the ALDEFLUOR™ Reagent, which is a fluorescent substrate for ALDH, with (control sample) or without (test sample) the specific ALDH inhibitor dimethylamino benzaldehyde (DEAB). The fluorescent reaction product, that is retained within the cells and is proportional to the activity of ALDH, was measured by flow cytometry (Attune® Acoustic Focusing Cytometer, Applied Biosystems). Data acquisition was performed using identical instrument settings for each test and control sample on a population of 20,000 cells in a SSC-H/BL1-H Dot Blot Diagram.

### Mammosphere formation

Mammosphere formation was assayed as previously described (32). For primary mammosphere generation, cells (70–80% confluent) were detached by trypsinization, centrifuged at 580 × g for 2 min, resuspended in 2 ml of ice-cold PBS and passed several times through a 25 G syringe needle. Then cells (600 cells/cm^2^) were seeded in 6-well plates, coated with 1 ml/well of 1.2% poly-2- hydroxyethyl methacrylate (pHEMA) solution in absolute ethanol. Cells were incubated, in the presence of APRIL or BAFF (100 ng/ml), testosterone-BSA (10^−6^M) or vehicle, in a humidified atmosphere at 37°C and 5% CO_2_, for 7–12 days, without disturbing the plates or replenishing the medium. The number of mammospheres (primary generation, defined as a cellular mass of at least 10 cells more than 50 μm in size) was counted, with a Leica DMIRE2 inverted microscope, at 400X magnification. For the generation of secondary mammospheres, primary mammospheres were collected, washed twice with 1 ml PBS, centrifuged at 115 g for 5 min and trypsinized with 300 μl of 0.5% trypsin/0.2% EDTA at 37°C for 2 min. After trypsin neutralization with 1 ml of serum-containing medium and centrifugation at 580 g for 5 min, single cells from were resuspended in 200 μl of ice-cold PBS and seeded in pHEMA coated 6-well plates (600 cells/cm^2^). Cells were incubated for 7 additional days and secondary mammospheres were counted as described above.

### Immunocytochemistry

For the detection of the expression of pluripotency markers in breast cancer cells, treated or not (control) with APRIL or BAFF (100 ng/ml) for 4 days, cells were fixed with 2% paraformaldehyde. Afterwards, they were incubated at room temperature for 1 h with 3% BSA in TBS, and then overnight at 4°C with primary antibodies (c-MYC 1/600, ALDH1A1 1/800, SOX-2 1/100 all from Santa-Cruz Biotechnology, and NANOG 1/100 from eBiosciences). For the detection of antibody binding, the UltraVision LP Detection System: HRP Polymer Quanto (Thermo Scientific, Cheshire, UK) was used, with 3'3-Diaminobenzidine (DAB) as chromogen. Stained cells were lightly counterstained with Harris hematoxylin for 10 s, hydrated and mounted in Permount (Fisher Scientific, Fair Lawn, NJ). Internal controls for specificity of immunostaining included replacement of primary antibody with non-specific serum (negative). Slides were evaluated for the presence and the intensity of staining (quantified using ImageJ).

### EMT transition

The EMT status of cells was determined by measuring the ratios of vimentin/keratins expression levels, using image analysis after laser scanning microscopy (33) and by flow cytometry. Cells in cytospin preparations (used for microscopy) and in suspension (used for microscopy and flow cytometry) were immunostained with primary antibodies for keratins 8, 18, 19 (mouse monoclonal antibodies, A45-B/B3, R002A, Micro- met AG, Munich, Germany) and vimentin (polyclonal antibody, Santa Cruz Biotechnology, sc-7558) and anti-mouse and anti-rabbit secondary antibodies labeled with Alexa 488 (Invitrogen) and CF555 (red staining, Biotium) dyes respectively. Untreated and treated cells from cytospin preparations and immunostained cells, attached on alcian blue coated coverslips, were analyzed by confocal (Leica SP) microscopy. To prevent any signal interference (green and red) generated by the different emission spectra, the detection of each marker was performed by sequential laser confocal scan. Fixed confocal settings were used for all specific measurements. Images were taken from 50 cells of each treatment and were stored electronically. To quantify the fluorescence intensity of vimentin and keratins, the images were subjected to Java-based image processing in Image J, and the ratio of red (vimentin) vs. the green (keratin) fluorescence intensity was calculated. In parallel, for verification purposes, cells immunostained in suspension were also analyzed by flow cytometry (Attune® Acoustic Focusing Cytometer, Applied Biosystems) at a cell population of at least 20,000 cells and the percentage of green and red labeled cells were obtained for each treatment condition.

### Wound healing assay

Cells were seeded in12-well plates and cultured until a monolayer was formed. At that point, cells were incubated for 1 h with mitomycin C (10 μg/ml), to prevent further cell proliferation. Afterwards, a thin wound was drawn in the middle of each well using a sterile micropipette tip and cells were washed with PBS in order to remove any remaining cell debris. Fresh medium containing BAFF or APRIL (100 ng/ml) with or without Testosterone-BSA (10^−6^ M), was added. The subsequent colonization of the denuded area was photographed with an inverted microscope (DM IRE2, Leica), at different time-intervals (at time 0, just before adding the tested agents 24–48 h afterward, see Results for details) always at predefined points of the well. The photographs were analyzed, and the covered distance was measured, and compared to the control cells.

### Actin cytoskeleton staining and visualization

Cells were grown on 8 -well chamber slides. After incubation with the different agents for 10 min, cells were washed with PBS twice, fixed with 2% paraformaldehyde in PBS for 10 min at room temperature, permeabilized with 0.5% Triton X-100 for 10 min and incubated in blocking buffer (2% BSA in PBS) for 15 min. Actin cytoskeleton was visualized by rhodamine-phalloidin staining (1:400 in PBS containing 0.2% BSA) for 45 min. Specimens were analyzed in a Leica SP confocal microscope.

### Knock down experiments (BCMA, JNK)

T47D cells were plated in six-well plates (2.3 × 10^5^ cells/well) and left to adhere for 24 h. The medium was changed, and transfection with siRNA for BCMA (NM_001192, Sigma-Aldrich) or shRNA against JNK1 or JNK2 prepared by our group, as reported in reference (19). Transfection was performed with a standard Lipofectamine 2000 protocol (Invitrogen; 0.8 mg DNA, 1 ml Lipofectamine 2000 in Optimem medium, for each well of a 24-well plate, scaling up for 6-well plates). Transfection efficiency was 85%, as estimated by counting GFP-positive cells.

### JNK inhibition

T47D cells were cultured in six-well plates and treated with APRIL or BAFF (100 ng/ml) for 4 days in the presence of the specific JNK inhibitor, SP600125 (10 μM).

### NFK-B activation assay

Cells were cultured in 24-well plate and were transfected with 0.2 μg/well of pNFκB-Luc plasmid (Clontech, Mountain View, CA), carrying NFκB response elements, in front of the 5′ end of the firefly luciferase gene, together with 0.2 μg/well of a Renilla luciferase vector (pRL-CMV, Promega, Fitchburg, WI), using Lipofectamine 2000 (Invitrogen, 1 ml per well) in Optimem medium. Cells were incubated for 24 h and then treated with BAFF or APRIL for 24 h. Luciferase activity was assayed with a Dual-Luciferase Reporter 1,000 Assay System (Promega, Fitchburg, WI), in a Berthold FB12 Luminometer (Bad Wildbad Germany).

### Statistical analysis

Statistics were performed by the use of SPSS v21 (SPSS/IBM Inc, Chicago, IL) and Prism v6.05 (GraphPad Inc, La Jolla, CA). Parametric tests were used, as appropriate and a two-sided *p* < 0.05 level was retained for significance.

## Results

### BAFF and APRIL expression in breast cancer cell lines—regulation by extranuclear-acting androgen

**Figure 1:**
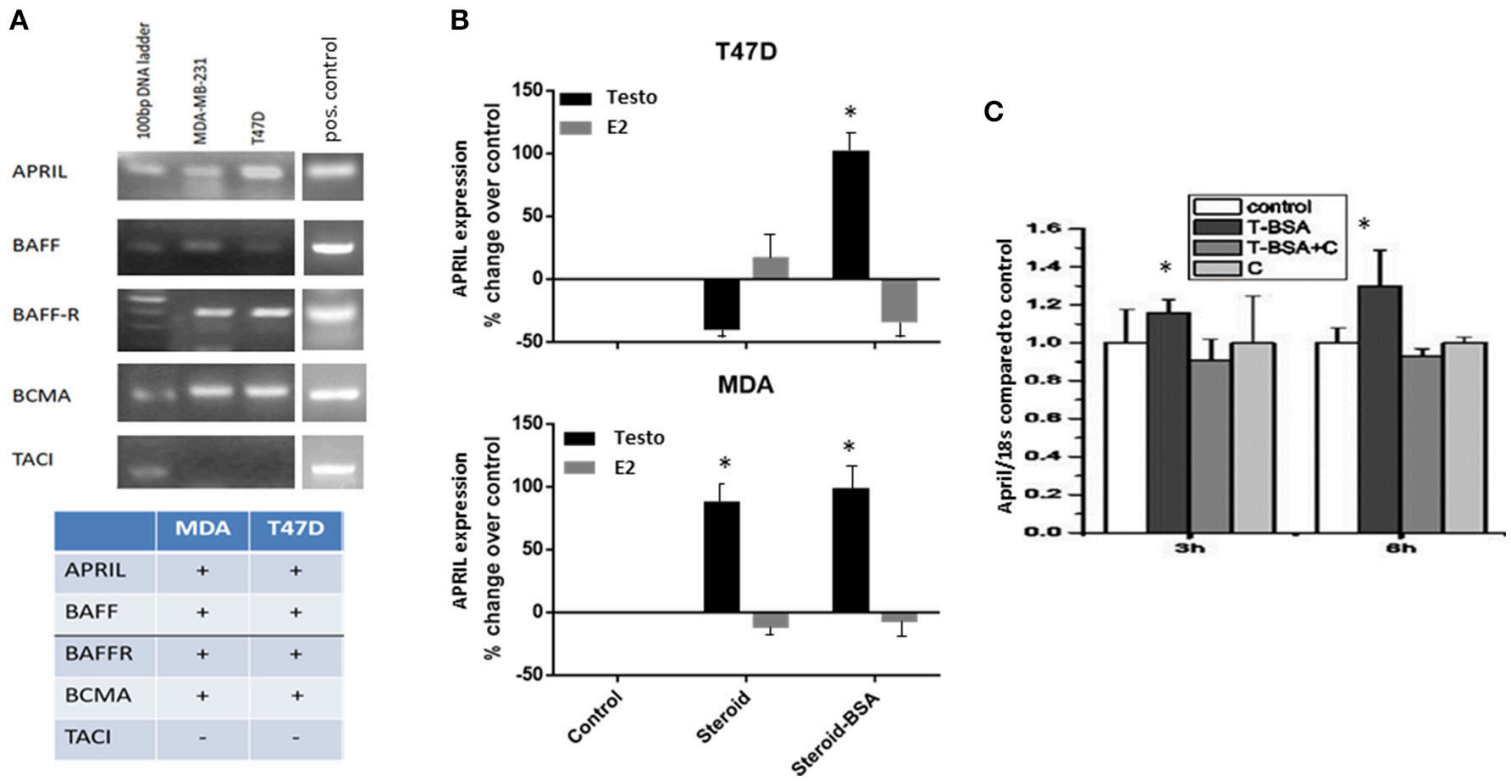
Expression levels of BAFF, APRIL, BAFF-R, BCMA, and TACI in breast cancer cell lines and the effect of steroid hormones on APRIL expression levels **(A)**. The expression of BAFF, APRIL, BAFF-R, BCMA, and TACI investigated by PCR in T47D and MDA-MB-231 cells breast cancer cell lines. Figure presents the results of a single experiment, while table shows the presence or absence of transcripts, based on three different experiments. See Material and Methods for further details and description of the controls. **(B)** APRIL expression [from data previously reported (20, 21)] and deposited in GEO under the numbers GSE18146, GSE32666, and GSE32668) after treatment of breast cancer cells for 3 h with steroid hormones, testosterone (Testo) or estradiol (E2), unconjugated or in their membrane impermeable form conjugated to BSA (steroid-BSA). See Material and Methods for details of data extraction and analysis. **(C)** Effect of testosterone-BSA (T-BSA) on APRIL expression (investigated by PCR) in T47D breast cancer cells in the presence or absence of cyproterone acetate **(C)** (*denotes *p* < 0.05).

We have assayed the presence of APRIL, BAFF and their receptors in an ERα-positive (T47D) and a triple negative breast cancer cell line (MDA-MB-231 cells, named thereafter MDA cells). BAFF, APRIL, BCMA, and BAFFR mRNA transcripts were present in both cell lines, while TACI transcript was constantly absent (Figure 1A).

To explore whether there is a relation between steroids and APRIL and BAFF production we have interrogated our previously reported transcriptome data on the effect of androgen (GSE18146) and estrogen (GSE32666 and GSE32668) on T47D and MDA cells (20, 21). As depicted in Figure 1B a significant increase of APRIL transcription was observed in MDA cells after 3 h-incubation with testosterone. Interestingly, when the membrane-only acting androgen (Testosterone-BSA) was used, this increase was observed both in T47D and MDA cells, suggesting an extranuclear androgen action. In contrast, estrogen had a minimal effect on APRIL transcription in both cell lines. Conversely, no significant modification of APRIL-BAFF receptors was found (not shown).

The above presented transcriptional activation of APRIL by membrane-acting steroids, was further explored by examining the possible modification of APRIL and BAFF transcription after cell incubation with the membrane-only acting, testosterone-BSA. Indeed, treatment of T47D cells with testosterone-BSA resulted in a significant increase of APRIL gene product at 3–6 h (Figure 1C). Interestingly, this effect was inhibited by the antiandrogen cyproterone acetate (which was ineffective *per se*). This result indicated that the effect of androgen might be mediated by membrane-bound AR and not by GPCR-related membrane-initiated androgen action, which is not modified by anti-androgen (34). In contrast, BAFF transcription was not modified (data not shown).

### BAFF and APRIL increase cell migration, epithelial-mesenchymal transition (EMT) and stemness in epithelial breast cancer cells

**Figure 2:**
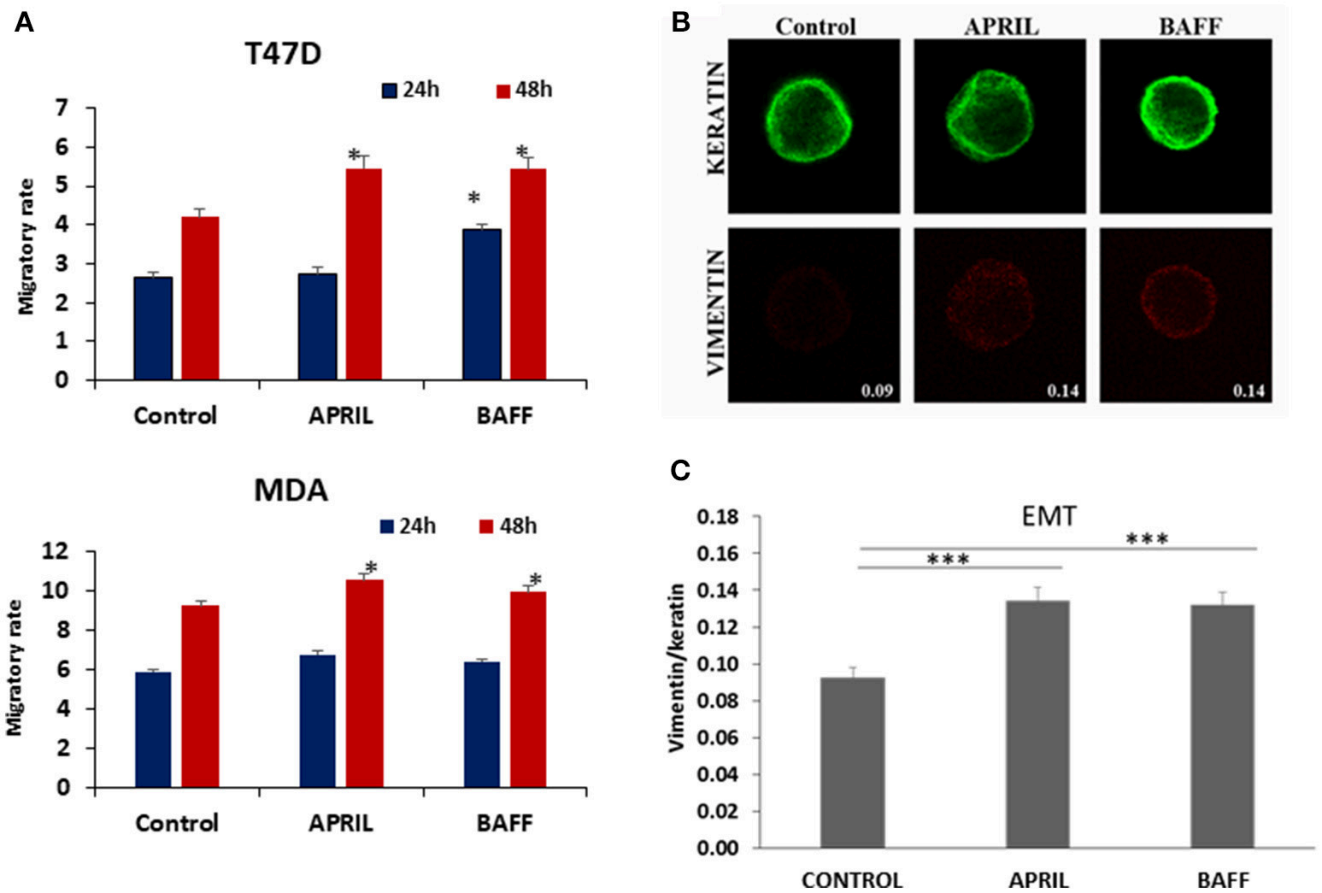
Effect of APRIL and BAFF on migration and EMT status**. (A)** Effect of APRIL and BAFF (100 ng/ml) on migration of T47D and MDA cells after 24–48 h of treatment. Figure presents mean ± SE of three independent experiments. (All samples were analyzed with *t*-test comparing at each time point, treatment vs. control, and *denotes *p* < 0.05) **(B)**. Representative photos (Magnification 1000x) of control (untreated) T47D cells and APRIL or BAFF (100 ng/ml) treated cells for 4 days immunostained in suspension for keratins (upper panel, green) and vimentin (lower panel, red). Fluorescence intensity was calculated using Image J. Values represent the ratio of vimentin/keratins expression in these cells. **(C)** Graphical presentation of EMT status changes obtained by calculating the ratio of vimentin/keratins expression of 50 cells for each treatment condition. All samples were analyzed with *t*-test (*** denotes *p* < 0.0001).

APRIL has been reported to be associated with increased breast tumor growth and metastasis (18). In our cell lines both APRIL and BAFF enhanced cell migration (the effect was more prominent after a 48 h-incubation, Figure 2A). Additionally, the EMT cell status of breast cancer cells was enhanced after treatment with either APRIL or BAFF (100 ng/ml), as depicted by an increase of vimentin/keratin staining ratio (Figures 2B,C). This was accompanied by actin cytoskeleton rearrangements, characteristic of migrating cells (Supplementary Figure 1), with the protrusion of filopodia. It is to note that APRIL and BAFF induce similar modification of EMT markers and migratory activity, suggesting an interaction occurring through the same receptor(s).

**Figure 3:**
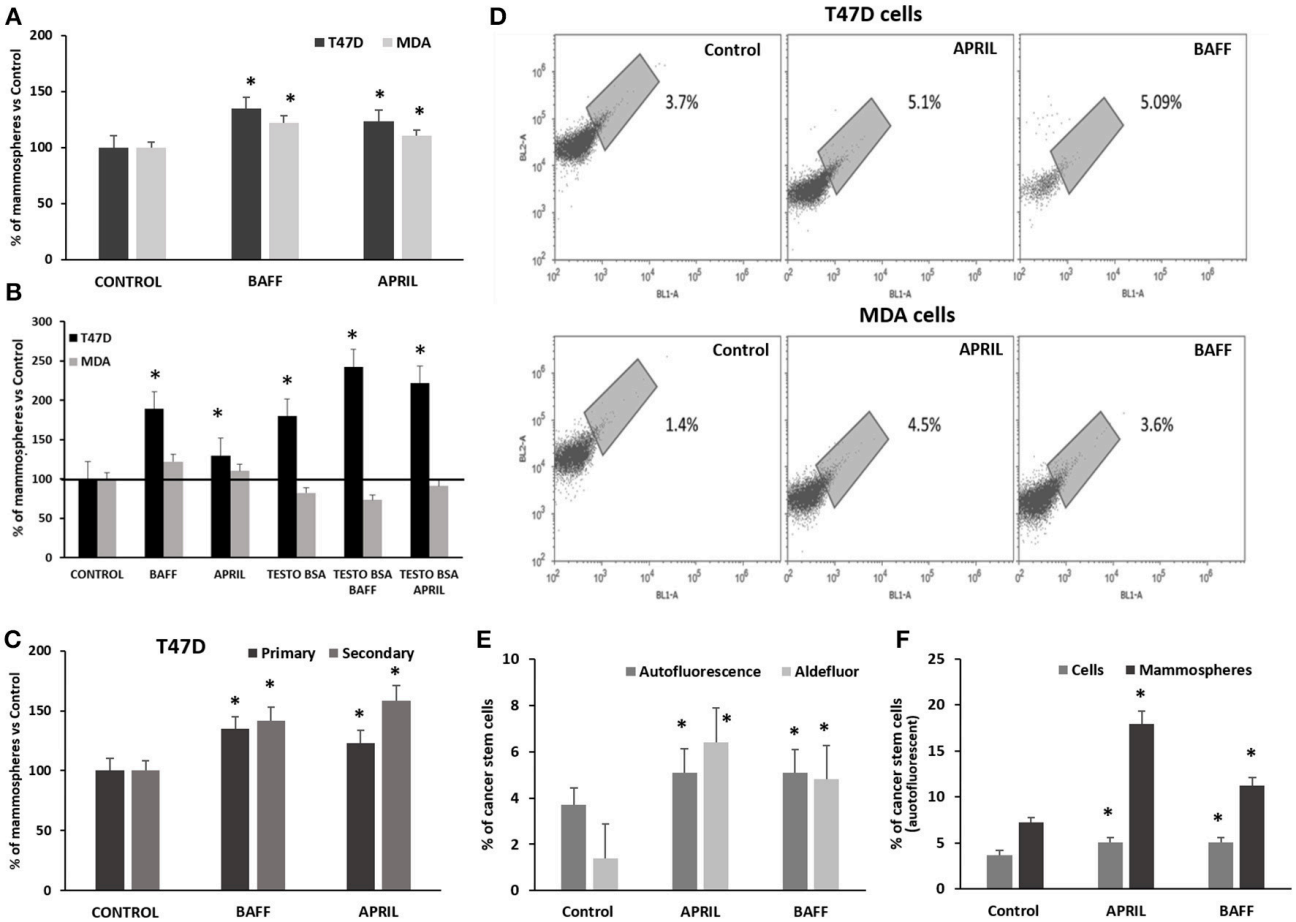
Effect of APRIL and BAFF on mammosphere formation and breast cancer stem cell population and of testosterone on the effect of APRIL and BAFF on mammosphere formation **(A)**. Percentage of primary mammospheres formed after treatment of breast cancer cells (T47D and MDA) with either BAFF or APRIL (100 ng/ml) for 9 days. Results of three independent experiments are expressed as a percentage of control (untreated) cells (mean ± SE, *denotes *p* < 0.05, at least). **(B)** Percentage of mammospheres (primary) formed after treatment of breast cancer cells (T47D and MDA) with either BAFF or APRIL (100ng/ml) in the presence or absence of testosterone-BSA, for 9 days. Results of three independent experiments are expressed as a percentage of control (untreated) cells performed (mean ± SE, *denotes *p* < 0.05). **(C)** Percentage of mammospheres formed after treatment of T47D cells with either BAFF or APRIL (100 ng/ml) for 9 days (primary mammospheres) compared to secondary mammospheres and a subsequent 7-day period. Results of three independent experiments are expressed as a percentage of control (untreated) cells. (mean ± SE, *p < 0.05) **(D)**. Percentage of breast cancer stem cells (T47D and MDA) with or without treatment with BAFF or APRIL (100 ng/ml) for 4 days as estimated by assaying them with high green autofluorescence on a population of 20,000 cells, in a BL2-A/BL1-A dot plot. Three independent experiments were performed and the results of a representative one is presented. **(E)** Comparison of the percentages of breast cancer stem cells as estimated by autofluorescence on a population of 20,000 cells in a BL2-A/BL1-A dot plot and ALDEFLUOR kit with or without treatment with BAFF or APRIL (100 ng/ml) for 4 days. Three independent experiments were performed (mean ± SE, *denotes *p* < 0.05). **(F)** Comparison of the percentages of breast cancer stem cells (identified by autofluorescence on a population of 20,000 cells in a BL2-A/BL1-A dot plot) in T47D cells and mammospheres treated or not with BAFF or APRIL (100 ng/ml) for 4 and 12 days respectively. Three independent experiments were performed (mean ± SE, *denotes *p* < 0.05).

In addition to EMT, another factor necessary for an increased metastatic potential and disease progression is a combination of genetic alterations and epigenetic events that recapitulate normal developmental processes, including stem cell self-renewal (35) and the acquisition of “stem cell” properties, through a “dedifferentiation” process. T47D and MDA cells, treated with APRIL or BAFF (100 ng/ml) exhibited an increased ability for anchorage-independent growth and the formation of mammospheres. More specifically, the number of primary mammospheres increased by 40% after 9 days of incubation (Figure 3A). The effect of these two agents was more prominent in ERα-positive T47D than in triple negative MDA cells. Interestingly, testosterone-BSA *per se* increased mammosphere formation in T47D cells. When cells were incubated with testosterone-BSA and APRIL, an additive effect was observed (Figure 3B). Focusing in the T47D cell line, in which mammosphere formation was more prominent, primary mammospheres were dispersed and cells were cultured under the same conditions (formation of secondary mammospheres). The effect of both cytokines was further enhanced, with APRIL being slightly more potent than BAFF (Figure 3C).

The effect of BAFF and APRIL on the induction of stemness was further verified by the intrinsic autofluorescence of epithelial cancer stem cells, due to riboflavin accumulation in membrane-bounded cytoplasmic structures, bearing ATP-dependent ABCG2 transporters (31). Incubation of breast cancer cells with APRIL or BAFF (100 ng/ml) for at least 4 days induced a significant increase in autofluorescence, from 2.5 ± 1.2% in control (untreated) T47D cells to 5 ± 0.5% in APRIL or BAFF-treated cells and from 2 ± 0.6% in control to 4 ± 0.5% in APRIL or BAFF-treated MDA cells (Figure 3D). This increase in autofluorescence correlates and is of similar amplitude with the increase of ALDH1A1 activity, assayed by flow cytometry and the ALDEFLUOR® kit (see Supplementary Figure 2 for an example and Figure 3E). Dispersed T47D cell mammospheres were assayed for a stem cell signature; they were found to have significantly more stem cells compared to cells from conventional monolayer culture (7.2 vs. 2.1%). BAFF or APRIL treatment further increased this autofluorescent stem cell signature (17.9 vs. 4.5 for APRIL and 11.2 vs. 3.6 for BAFF respectively) suggesting that these cytokines have the capacity to induce pluripotency in T47D cells (Figure 3F).

**Figure 4:**
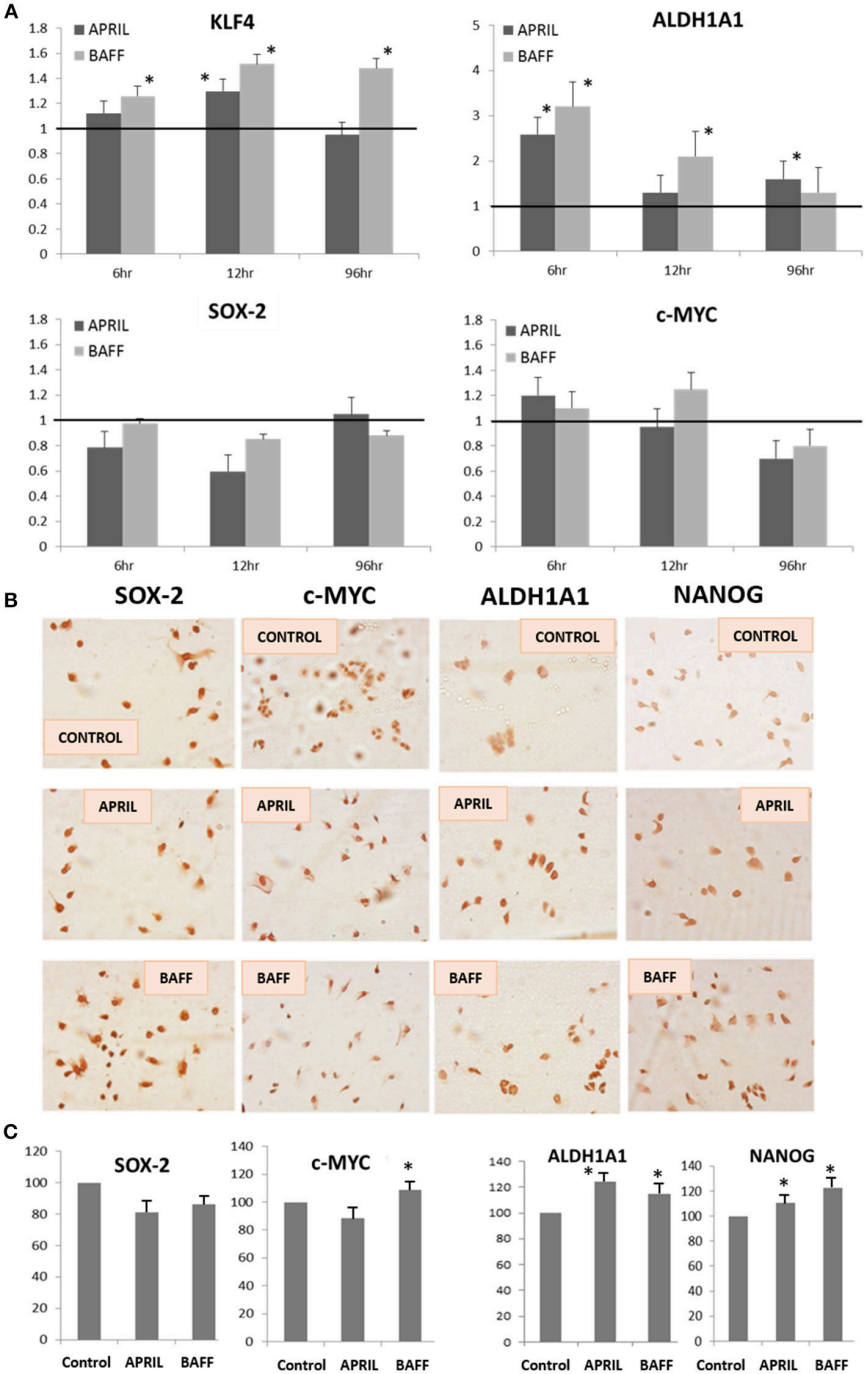
Expression levels of pluripotency markers in T47D cells after APRIL or BAFF treatment**. (A)** Expression levels of ALDH1A1, KLF4, SOX-2, and c-MYC, genes (quantified by Real-Time PCR and expressed as percentage of control) after treatment of T47D cells with APRIL or BAFF (100 ng/ml) for 4 days. Three independent experiments were performed (mean ± SE, *denotes *p* < 0.05, at least). **(B,C)** Expression of SOX-2, c-MYC, ALDH1A1, and NANOG proteins (assayed by immunocytochemistry). Representative images of three independent experiments are presented **(B)** Quantification of the proteins' expression levels was performed in at least 10 cells in three different photos for both control and treated cells using Image J and is expressed as percentage of control (mean ± SE, **(C)**).

To further verify the emergence of stemness after 4 days incubation with APRIL or BAFF (100 ng/ml), the transcription of pluripotency-related factors (SOX2, c-MYC, KLF4, NANOG, and ALDH1A1) was investigated in T47D cells. The expression of the above genes was examined both at the mRNA (by Real Time PCR, Figure 4A) and protein level (by immunocytochemistry, Figures 4B,C). As shown, APRIL and BAFF mainly increase the expression of ALDH1A1, KLF4, and NANOG. Similar data were obtained in MDA cells (Supplementary Figure 3). Furthermore, we analyzed published data (22) from a microarray comparing the transcription profile of 56 breast cancer cell lines. A significant positive correlation was observed (Supplementary Figure 4) for APRIL (TNFSF13), BAFF (TNFSF13B), and BCMA (TNFRSF17) with the androgen receptor (AR) with *p*-values 0.00007, 0.002, and 0.002 respectively. In addition, a significant positive correlation between APRIL (TNFSF13) and BCMA (TNFRSF17), SOX2 or KLF4 (*p* = 0.018, 0.021, and 0.012 respectively) was found. Contrariwise, no correlation between APRIL and Myc or NANOG were observed (not shown).

### APRIL and BAFF modulate stemness markers expression through BCMA and JNK signaling

**Figure 5:**
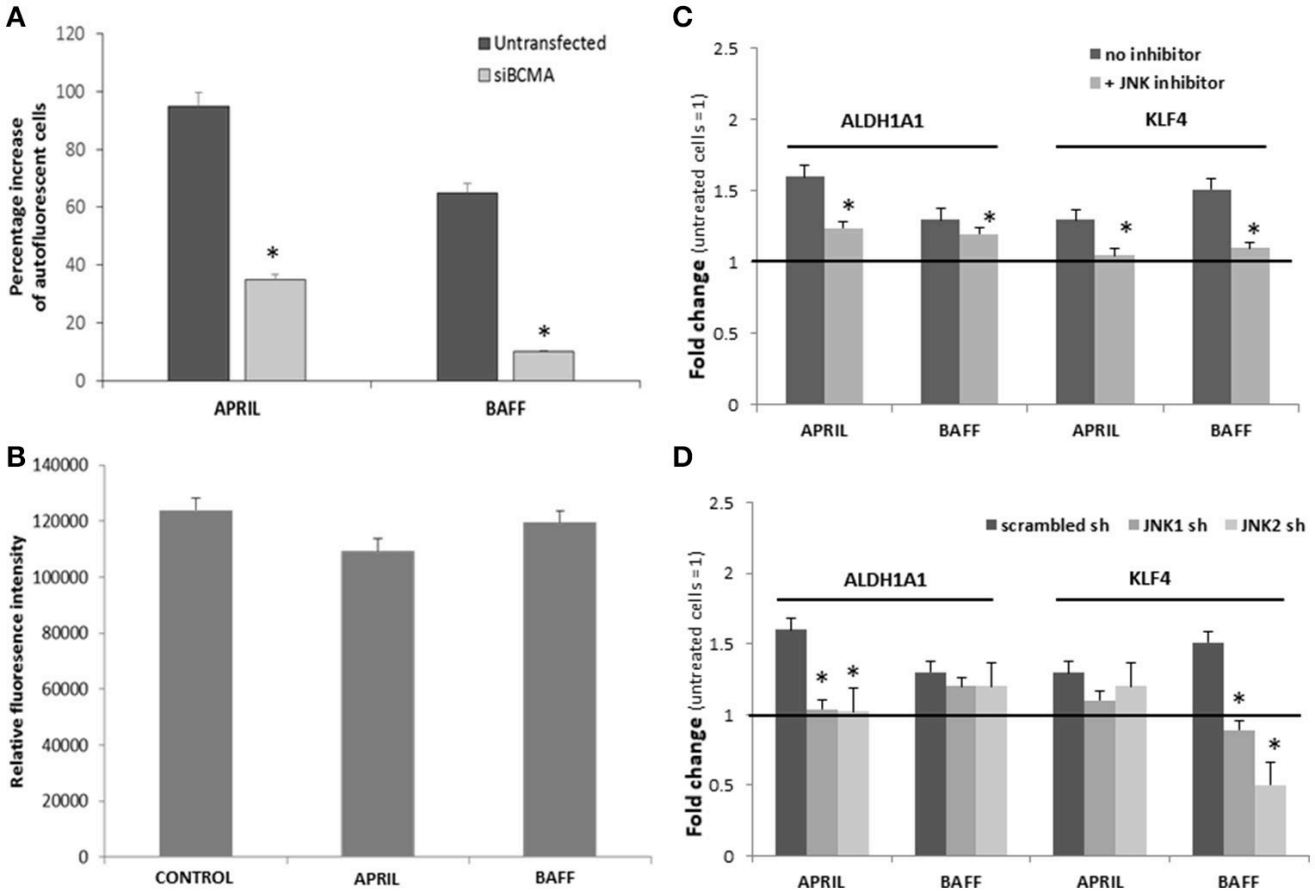
**(A)** Effect of APRIL and BAFF on breast cancer stem cells in the presence of siBCMA. Percentage increase in the number of breast cancer stem cells transfected or untransfected with siBCMA and treated with BAFF or APRIL (100 ng/ml) for 4 days. (Breast cancer stem cell number was estimated by counting the cells with high green autofluoresence in a BL2-A/BL1-A dot plot). **(B)** NFk-B activity of untreated (control) and treated with APRIL or BAFF (100 ng/ml, for 24h) T47D cells. Experiments were performed in triplicate. **(C,D)** Effect of APRIL and BAFF on ALDH1A1 and KLF4 expression in the presence of a JNK inhibitor or sh RNA for JNK1 or JNK2. Expression levels of ALDH1A1 and KLF4 after treatment of T47D cells with APRIL or BAFF (100 ng/ml) for 4 days in the presence of a JNK inhibitor SP600125 (10 μM) **(C)** or sh RNA for JNK1 or JNK2 **(D)**. Data are presented as a mean ± SE of three independent experiments (*denotes *p* < 0.05, at least).

Results presented so far suggest that APRIL and BAFF promote in a similar way the emergence of pluripotency in breast cancer cells. Taking into consideration that T47D cells express BCMA, on which both cytokines can bind, and BAFFR, an exclusive BAFF receptor (12) (see Figure 1A), we assumed that this effect might be mediated by BCMA. To confirm this hypothesis, we transfected T47D cells with a specific anti-BCMA siRNA. Transfected cells were then incubated with BAFF or APRIL and pluripotent/stem cells were assayed by their autofluorescence. As depicted in Figure 5A, cancer stem (autofluorescent) cells were significantly decreased, suggesting the mediation of this effect through BCMA. To further explore the underlying signaling, we have investigated the two pathways mediating post-receptor effects of BAFF and APRIL [TRAF signaling and NFκB activation (36), and BCMA-specific signaling, mediated by JNK (37)]. As shown in Figure 5B, T47D cells, transfected with a specific plasmid, carrying NFκB response elements in front of the firefly luciferase gene, do not show any activation after a 24 h BAFF or APRIL incubation; contrariwise, the JNK specific inhibitor, SP600125 or shRNAs against JNK1 or JNK2, significantly blocked the effects of APRIL and BAFF on ALDH1A1 and KLF4 (Figures 5C,D), indicating that, in breast cancer cells, APRIL and BAFF, binding to BCMA, signal toward pluripotency *via* JNK1 and JNK2.

### APRIL in patients' samples

**Figure 6:**
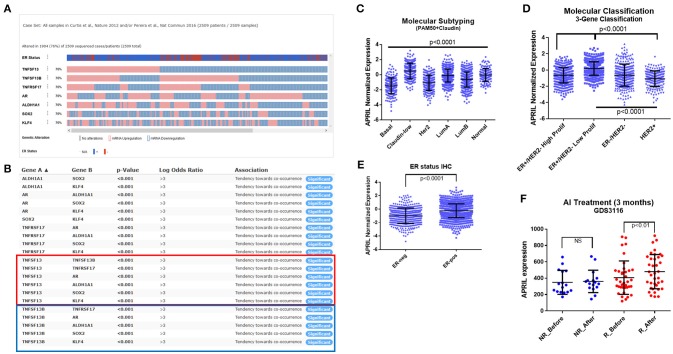
APRIL expression in breast cancer specimens. *Analysis of the MetaBRIC* (23, 24) *study* **(A-E)**, in the cBioPortal (25–27) web environment. **(A)** Normalized expression of APRIL (TNFSF13), BAFF (TNFSF13B), BCMA (TNFRSF17), androgen receptor (AR) and the pluripotency genes ALDH1A1, SOX2, and KLF4. The IHC ER positive (red) and negative (blue) cases is also depicted. **(B)** Co-expression analysis of the genes presented in A, performed in c-BioPortal. The red box presents APRIL and the blue one BAFF co-occurrence analysis. **(C–D)**. Analysis of molecular classification of the MetaBRIC study for normalized APRIL expression, according to the PAM50+claudin and the 3-gene metagene. **(E)** Analysis of normalized APRIL expression in IHC ER status of the MetaBRIC patients. **(F)** *Analysis of APRIL expression in the letrozole GDS3116 study* (28, 29), according to the patients' response to a 14 days-treatment. Data from non-responders (NR, blue) and responders (R, red) are depicted, before and after letrozole treatment. See Material and Methods and Results sections for additional details. Data in panels **(C–F)** are analyzed by GraphPad Prism and depicted as individual values and mean ± SD. Statistical analysis was performed by ANOVA with *post-hoc* Turkey test in panels **(C,D,F)**, and Student's T-test in panel **(E)**.

As shown above, androgen enhances APRIL production in breast cancer cells regardless of estrogen receptor status. We hypothesized that increased local androgen (as could occur in patients treated with aromatase inhibitors that inhibit conversion of local androgen to estrogen), could enhance APRIL production, ultimately increasing cancer pluripotency. First, by analyzing a large breast cancer study (MetaBRIC study), we found that APRIL (TNFSF13), BAFF (TNFSF13B), and BCMA (TNFRSF17) were expressed in 76% of the studied cohort (Figure 6A). APRIL (red box in Figure 6B) and BAFF (blue box in Figure 6B) were significantly (*p* < 0.01) co-expressed with BCMA (TNFRSF17), AR and the pluripotency genes ALDH1A1, SOX2, and KLF4, confirming our data in breast cancer cell lines. Furthermore, APRIL was significantly higher in claudin-low and Luminal A tumors, detected by a PAM50+claudin metagene (Figure 6C) and significantly lower in Her2 positive tumors, identified with the PAM50 and a three-gene classifier (Figures 6C,D). Finally, as expected, ER-positive tumors, identified by immunohistochemistry, have a higher APRIL transcript content (Figure 6E).

Unfortunately, in the MetaBRIC study, we could not identify patients treated specifically by aromatase inhibitors. However in GDS3116 study (28, 29) paired transcriptome data of 53 letrozole (an aromatase inhibitor, AI) treated patients were included, prior and 14 days following AI therapy, together with their response to treatment (69.8% responded to the therapy, while 30.1% were non-responders after 3 months). In responders, in which a higher local testosterone concentration is expected, due to the action of AI, APRIL transcripts (Figure 6F, red dots) are higher after letrozole treatment. In contrast, in non-responders (Figure 6F, blue dots) APRIL transcripts remain unaltered. Further analysis for transcription factor modifications through gene transcript changes [using Web resource ISMARA (30)], revealed that, after Letrozol treatment, E2F family of transcription factors is decreased, while members of the C/Ebp and Fox families (FOXD, FOXO) are increased. Interestingly, all these transcription factors are tethered members of AR regulation (38), while C/Ebp factors were found to increase the activity of the APRIL promoter (*z* = 2.5). This result provides a proof of concept about the *in vivo* modification of APRIL transcripts, in breast cancer patients, by local androgen concentrations, induced by hormone-modification therapy.

## Discussion

Our understanding of breast cancer physiology has expanded in recent years. In addition to the molecular analysis and identification of different breast cancer subtypes (39, 40), recent data revealed a biological evolution of breast cancer cells from the primary to the metastatic site of the disease, influencing the disease progression (41), identified as a possible novel therapeutic target (42). A major element of breast cancer molecular and phenotypic evolution is the differentiation of stem cells from their niches (2, 5, 41) and their cross-talk with the epithelial, stromal and immune components of the niche or surrounding microenvironment (1, 4, 5, 41–43). However, another element of this heterogeneity has been recently identified and is related to the effects of administered treatment to the transformation of cancer epithelial cells (44), including the emergence of cell pluripotency. Recently, we have reported that short term exposure of breast cancer cells to tamoxifen may act as such an epigenetic stem-inducing stimulus (45). In the present work we provide evidence that an increase in breast cancer stem population can be induced by the two TNF superfamily cytokines BAFF and APRIL through a BCMA-JNK pathway. Moreover, patient data analysis revealed that this could be a possible mechanism of therapy resistance, since high APRIL levels may occur as a result of androgen accumulation, in aromatase inhibitors-treated patients.

The various markers currently used for breast cancer stem cells (BCSCs) (46, 47) identify slightly different subpopulations and it is, therefore, necessary to utilize more than one specific marker and/or tests for cancer stem cell properties (48). Therefore, in the present work, to investigate the effect of APRIL and BAFF, we utilized multiple approaches to detect BCSCs, including the expression at the mRNA and protein level of pluripotency markers, primary and secondary mammosphere formation, ALDH1A1 activity and the autofluorescence of BCSC. Our results show that both BAFF and APRIL increase the percentage of breast cancer stem cells by a two to three-fold, after at least 4 days of treatment. This finding suggests that the epigenetic changes required for the induction of this phenotype need a prolonged exposure to these agents. In fact, breast cancer cells, treated with APRIL or BAFF, display a more aggressive phenotype, as they exhibit: (1) increased migration and parallel modifications of the actin cytoskeleton; (2) increased expression of mesenchymal markers and signs of the acquisition of a mesenchymal phenotype. Such a phenotype is linked to stem-like cells (49, 50) and is associated with metastasis and a higher migratory capacity (51, 52); (3) increased stem cell population, as assayed by breast cancer stem cell autofluorescence and ALDH1A1 activity, and (4) increased mammosphere formation, with a significant increase in stem cell population. These results are in accordance with previously reported findings on APRIL promoting breast cancer lung metastasis (18), and our finding analyzing the MetaBRIC (23, 24) study, that APRIL and BAFF are co-expressed with pluripotency-related genes and that APRIL is preferentially expressed in claudin-low tumors.

An important finding supporting the observed stem cell enrichment after treatment with APRIL or BAFF is the significant acquisition of pluripotency markers KLF4, c-MYC, SOX2, and ALDH1A1 (53–56), at the mRNA level, in a sustained or transient manner and an increase of ALDH1A1 and NANOG in immunocytochemistry, after 4 days of incubation with APRIL or BAFF. It is to note that NANOG expression is the final step of pluripotency markers, related to the activation of the transcription factor network c-MYC-SOX2-Oct-KLF4 cascade (54, 57, 58). For this reason, we have chosen to detect NANOG protein in immunocytochemistry, as a final marker of pluripotency. These markers may act as transcription factors that promote a stem cell phenotype, maintain pluripotency and prevent differentiation. The fact that, in the studied cell lines, both BAFF and APRIL induced similar effects on these pluripotency markers at the mRNA and the protein level suggests that their action should be mediated through BCMA, a cognate receptor for both ligands (12). This was further supported by the inhibitory action of the specific anti-BCMA siRNA on their effects and the fact that the BCMA specific signaling involving JNK activation was found to be involved. Contrariwise, no NFκB activity was detected. Therefore, it seems that, in breast cancer cells, APRIL-BCMA signal toward pluripotency is mediated by JNK, a signaling pathway also reported previously by our group to mediate the effect of APRIL in hepatocellular carcinoma cell lines (19). The complete inefficiency of BAFF to modify NFκB, further suggests that, in our settings, BAFFR signaling, might be ineffective in this cell line. Moreover, we report that this APRIL-BCMA regulated effect relies on local androgen. Indeed, by analyzing our previously reported transcriptome data on T47D and MDA cells (17, 21, 59), we report that APRIL transcription was significantly increased after a 3 h-incubation with testosterone, while estradiol had only a minimal effect. Interestingly, a significantly higher change was induced by membrane-only acting testosterone (Testosterone-BSA), suggesting a membrane-initiated androgen action, while the inhibition of the effect by anti-androgen suggests that this effect might be mediated by the classical androgen receptor, activated at the membrane level.

This androgen-induced APRIL production and subsequent stemness, could possibly have a clinical importance in the case of breast cancer patients that are treated with aromatase inhibitors (AI), which can result in an increased local androgen concentration. To provide a proof of principle of this hypothesis, we extracted APRIL data from a series of letrozole-treated patients (28, 29). APRIL expression was significantly higher in the responder's group (expected to have increased local androgen levels), while it was not changed in the non-responders, verifying our hypothesis on testosterone-control of APRIL in breast cancer. Interestingly, in AI-responders, transcription factors related to a tethered AR effect (38) were found modified, while one of them (cEbp group) induced an increase of APRIL promoter activity.

Our study presents some limitations. First, only two breast cancer lines are tested. However, analysis of 56 breast cancer cell lines (22) and the clinical studies, presented above, provide a degree of validation of our findings. Our -omics approaches based on a retrospective analysis of publicly available data also supports our results. Future prospective studies could provide more refined data to the mechanism presented here. As aromatase inhibition is widely used to treat ER-positive tumors and AR profiling is part of cutting-edge approaches in breast cancer, the implications of a possible androgen-APRIL/BAFF-pluripotency enhancement might be of significant clinical value in providing a beneficial personalized therapy to patients.

In conclusion, our findings clearly indicate that both BAFF and APRIL can increase the percentage of breast cancer stem cells through BCMA-JNK mediation, pointing out for the first time that APRIL and BAFF not only modify breast cancer cell proliferation, but they can also contribute to the re-formation of the tumor. Additionally, they provide evidence for a new possible mechanism of therapy resistance that involves increased stemness by high APRIL levels, because of the accumulation of androgen that could occur in aromatase inhibitors-treated patients. They further provide evidence that, in order to establish optimal personalized, immune-related therapies, in breast cancer patients, one should, in addition to the targeting of the stroma and cancer-infiltrating immune cells (8), also investigate and target tumor cells, as was recently reported for another member of the TNFRSF, TNFR2 (10, 11).

## Author contributions

VP, GN, AT, EC, and MK conceived and designed the experiments and wrote the paper. VP, GN, PA, KA, FK, NP, KK, EK, HP, and PT performed the experiments and analyzed the data. All authors approved the manuscript.

### Conflict of interest statement

The authors declare that the research was conducted in the absence of any commercial or financial relationships that could be construed as a potential conflict of interest.
