# Supplementary material for: BCMA (TNFRSF17) Induces APRIL and BAFF Mediated Breast Cancer Cell Stemness
Source: Front Oncol. 2018 Aug 7;8:301. doi: 10.3389/fonc.2018.00301 (PMC6091000; doi:10.3389/fonc.2018.00301)
Supplement: Supplementary file 1 [file Data_Sheet_1.docx]

**Supplementary Figures**

**Supplementary Figure 1**

**
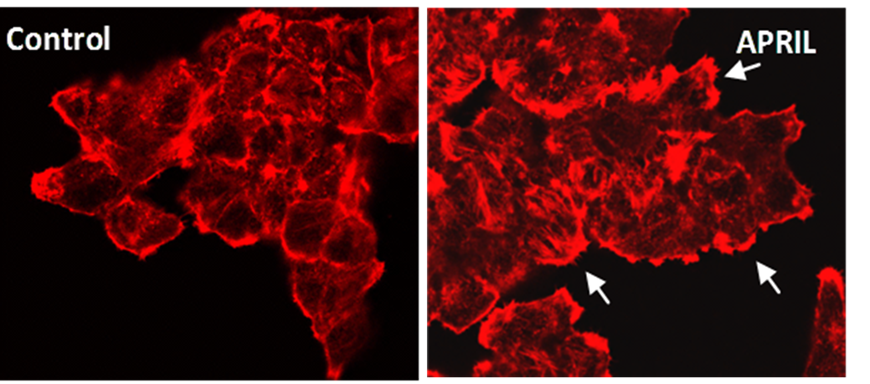
**

**Representative images of actin cytoskeleton of T47D cells treated with APRIL.** Actin cytoskeleton was visualized by rhodamine-phalloidine staining and a representative image of untreated (control) and treated with APRIL (100ng/ml, for 20 min) T47D cells are presented. Arrows point out fillopodia. Experiments were performed in triplicate.

**Supplementary Figure 2**


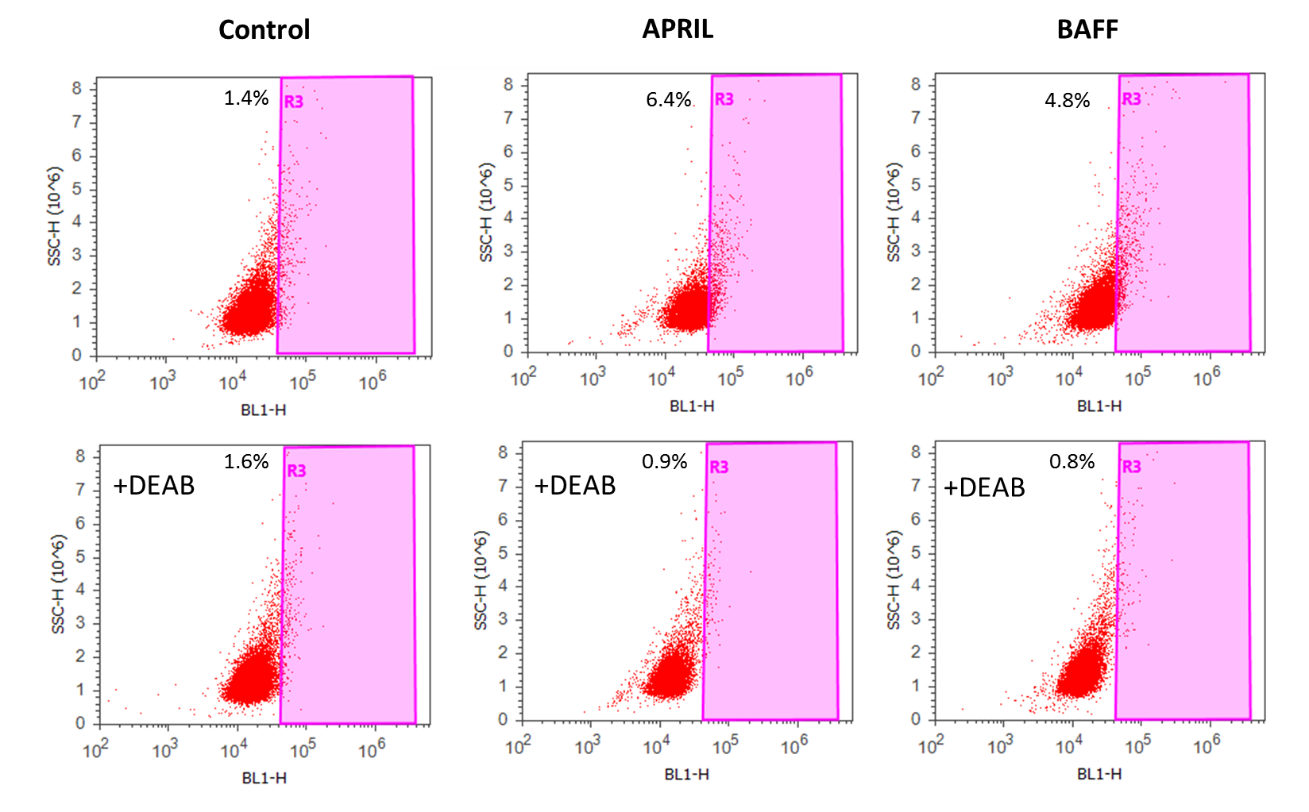


**Percentage of breast cancer stem cells that are positive for ALDH activity as estimated by the ALDEFLUOR kit.** Cells were treated with or without APRIL or BAFF (100ng/ml) for 4 days. Lower panel presents DEAB-treated cells that serve as the negative control, since DEAB is an inhibitor of ALDH1A1 activity. Three independent experiments were performed and the results of a representative one are presented.

**Supplementary Figure 3**


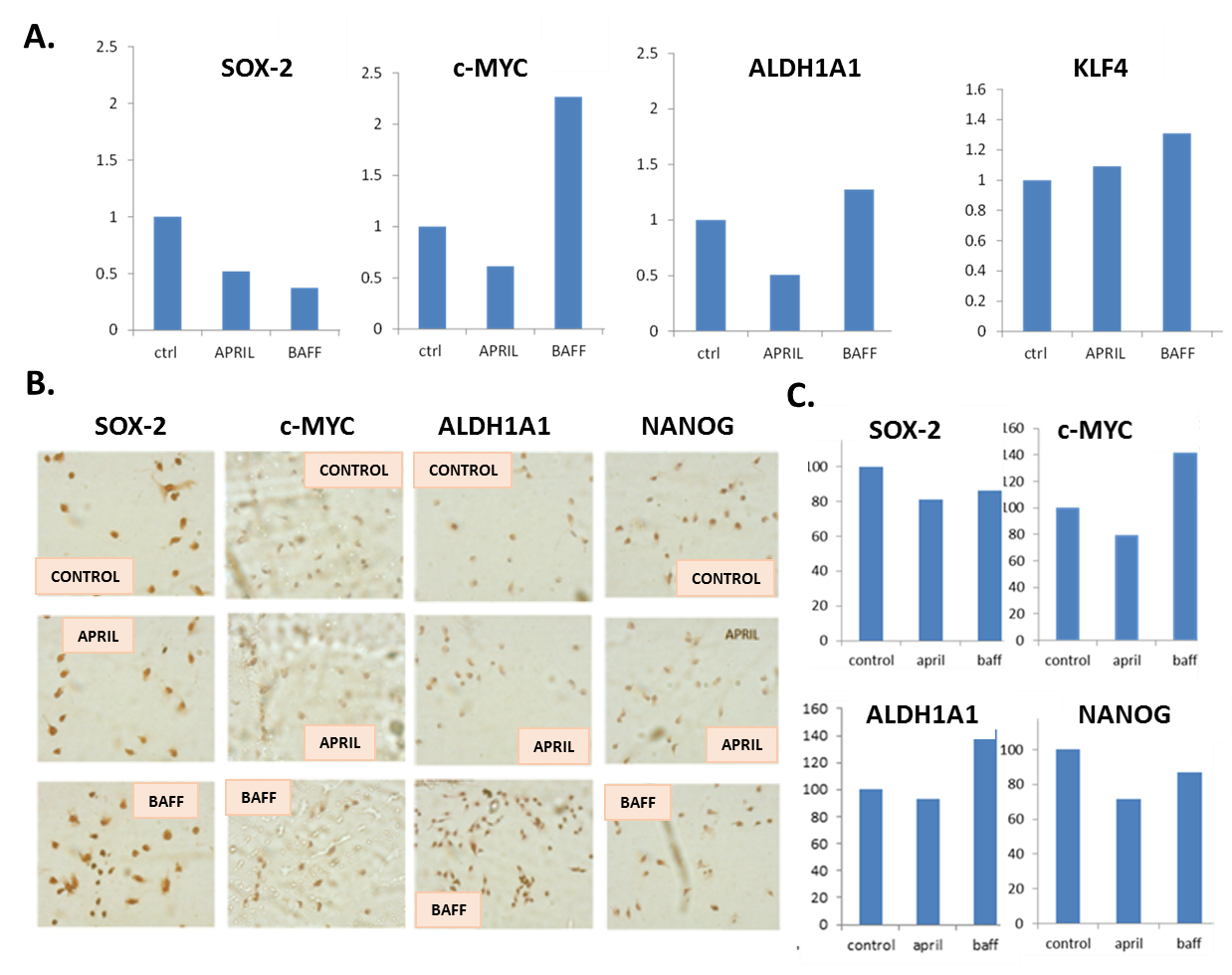


**Expression levels of pluripotency markers in MDA cells after** **APRIL or BAFF treatment.** **A.** Expression levels of ALDH1A1, KLF4, SOX-2, and c-MYC, genes (quantified by Real-Time PCR and expressed as percentage of control) after treatment of MDA cells with APRIL or BAFF (100ng/ml) for 4 days. **B. and C.** Expression of SOX-2, c-MYC, ALDH1A1 and NANOG proteins (assayed by immunocytochemistry). Representative images of three independent experiments are presented **(B)** Quantification of the proteins’ expression levels was performed using Image J and is expressed as percentage of control **(C).**

**Supplementary Figure 4**

**
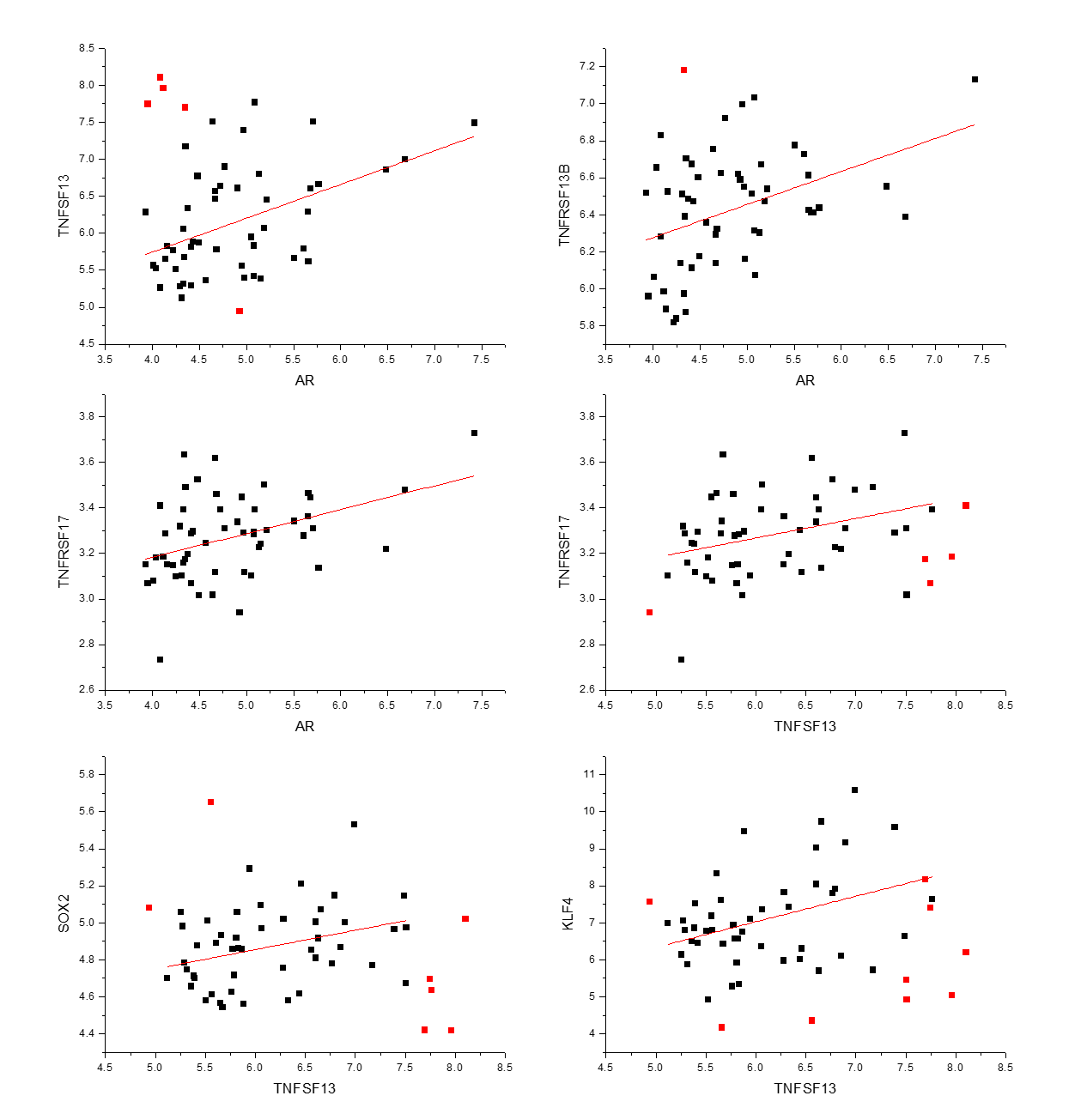
**

**Correlation analysis of APRIL (TNFSF13), BAFF (TNFSF13B), BCMA (TNFRSF17), androgen receptor (AR), SOX2 and KLF4 in 56 different breast cancer cell lines.** Normalized matrix data of the study E-TABM-157 were downloaded from *Array Express*. Expression data for TNFSF13, TNFSF13B, TNFRSF17, AR, SOX2, ALDH1A1, c-Myc, KLF4 and NANOG were extracted and analyzed in Origin 8, through a linear fit. Outliers (the same in each analysis) were denoted in red.
